# Retraction Note: The antihypertensive effect of the coadministration of exercise and eugenol in deoxycorticosterone acetate-induced hypertension in rats

**DOI:** 10.1007/s00210-025-04756-x

**Published:** 2025-10-20

**Authors:** M. A. Abdallah, Essam Omar Ibrahem, Eman A. Ali, Sara G. Tayel, Reda A. A. Abo-Elsoud

**Affiliations:** 1 Medical Physiology Department, Faculty of Medicine, Menoufia University, Shebin El Kom, Menoufia Egypt; 2 Clinical Pharmacology Department, Faculty of Medicine, Menoufia University, Shebin El Kom, Menoufia Egypt; 3 Anatomy and Embryology Department, Faculty of Medicine, Menoufia University, Shebin El Kom, Menoufia Egypt; 4 Medical Physiology Department, Faculty of Medicine, Menoufia National University, Tukh Tanbisha, Birket ElSaba, Menoufia Egypt; 5 Clinical Pharmacology Department, Faculty of Medicine, Menoufia National University, Tukh Tanbisha, Birket ElSaba, Menoufia Egypt; 6 Anatomy and Embryology Department, Faculty of Medicine, Menoufia National University, Tukh Tanbisha, Birket ElSaba, Menoufia Egypt

**Retraction Note: Naunyn-Schmiedeberg's Archives of Pharmacology (2025) 398:14041–14056**

10.1007/s00210-025-04119-6

The Editor-in-Chief has retracted this article. Following publication, the Editors have noted multiple overlaps within and between different panels in Figure 7. The authors were contacted for an explanation and raw images regarding concerns in Figure 7. The authors failed to provide a satisfactory explanation, and the provided raw images do not match the published images for Figure 7.

The Editor-in-Chief therefore has lost confidence in the results presented in this study.

All authors disagree with this retraction.

